# Kikuchi–Fujimoto disease associated with the central nervous system in children

**DOI:** 10.1002/pdi3.23

**Published:** 2023-08-14

**Authors:** Yuyao Huang, Xinpeng Wang, Ling Wang, Juan Wang, Chengbing Tan, Min Cheng, Yan Jiang, Li Jiang

**Affiliations:** ^1^ Department of Neurology Children's Hospital of Chongqing Medical University National Clinical Research Center for Child Health and Disorders Ministry of Education Key Laboratory of Child Development and Disorders Chongqing Key Laboratory of Translational Medical Research in Cognitive Development and Learning and Memory Disorders Chongqing China

**Keywords:** central nervous system, children, histiocytic necrotizing lymphadenitis, Kikuchi–Fujimoto disease, Kikuchi necrotizing lymphadenitis

## Abstract

The clinical data of patients with Kikuchi–Fujimoto disease (KFD) combined with the involvement of the central system is limited, particularly for children. This study aimed to investigate the clinical features and prognosis of KFD associated with the central nervous system in children. We collected the clinical data of patients diagnosed with KFD combined with the involvement of the central system, hospitalized at Children's Hospital of Chongqing Medical University (CHCMU). Furthermore, we summarized the clinical features and outcomes in children by reviewing the published literature. Together with the two patients from CHMCU enrolled in this study, we identified 19 children diagnosed with KFD combined with the involvement of the central nervous system, including eight males and 11 females. The onset age ranges from 6 to 18 years (the median age was 14 and the interquartile range was 9–16). Sixteen patients were diagnosed with aseptic meningitis. Headache (78.9%, 15/19) was the most common symptom. Cerebrospinal fluid pressure was increased in 3 patients (60.0%, 3/5), cell count was elevated in 14 patients (73.7%, 14/19), glucose was decreased in 4 patients (21.0%, 4/19), and protein was elevated in 12 patients (63.1%, 12/19). A total of 9 patients had cranial imaging changes. Electroencephalogram (EEG) was performed on seven patients and 4 patients presented abnormal EEG (57.1%, 4/7). A total of 12 patients received steroid therapy; two were combined with antiepileptic treatment. In children, KFD complicated with the involvement of the central nervous system was rare and aseptic meningitis was the primary manifestation. Most patients had a good prognosis and a few had residual sequelae of neurological damage.

## Graphical abstract

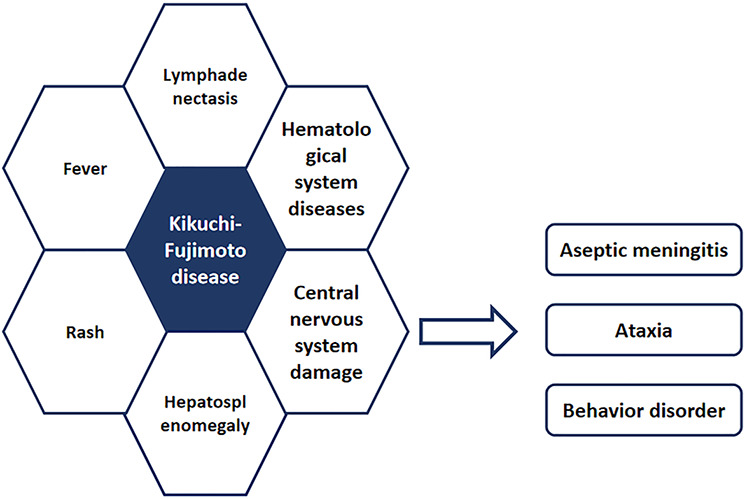

## 1 INTRODUCTION

Kikuchi–Fujimoto disease (KFD), also known as histiocytic necrotizing lymphadenitis, is an inflammatory immunoreactive non‐neoplastic lymphadenopathy. The course of the disease is benign and self‐limited with clinical manifestations, including fever, lymphadenopathy, rash, hepatosplenomegaly, central nervous system (CNS) symptoms, and hemophilic cell syndrome.^1^, ^2^ In terms of the nervous system, KFD can cause damage to meninges, brain parenchyma, and peripheral nerves, and even neurological symptoms as prominent clinical manifestations or initial symptoms. Central nervous system involvement is rarely reported as the first symptom of this disease. These patients are prone to misdiagnosis. We selected 2 cases of KFD complicated with central nervous system damage admitted to the Children's Hospital of Chongqing Medical University (CHCMU) from 2010 to 2022. Furthermore, we retrieved information on all pediatric patients diagnosed with KFD with central nervous system involvement from PubMed to discuss whether the clinical manifestations, auxiliary examination, diagnosis, prognosis, and treatment of the disease were consistent and to investigate the clinical manifestations and prognosis of children with KFD.

## 2 METHODS

This study is a retrospective chart review at the Children's Hospital of Chongqing Medical University (CHCMU). Two patients hospitalized at the Children's Hospital of Chongqing Medical University (CHCMU) from 1 January 2010 to 1 July 2022 were selected for this study. From the targeted literature, we summarized the epidemiological and clinical characteristics, laboratory features, brain MRI, electroencephalogram, prognosis, treatment, and outcomes of 19 pediatric patients. The literature search was carried out in PubMed (www.ncbi.nlm.nih.gov/pubmed), Web of Science (www.webofscience.com), MEDLINE(www.nlm.nih.gov/medline), and Cochrane (www.cochrane.org). The search deadline was January 4, 2023 with the keywords: Kikuchi–Fujimoto disease, Kikuchi necrotizing lymphadenitis, KFD, histiocytic necrotizing lymphadenitis, HNL, and central nervous system. Articles were written in English and manually searched to select pediatric patients (0–18 years of age) diagnosed with Kikuchi–Fujimoto disease with the involvement of the central nervous system and extract relevant information focused on clinical manifestations, follow‐up time, and prognosis. KFD is diagnosed by performing an excisional biopsy of affected lymph nodes or fine‐needle aspiration cytology (FNAC) with typical clinical features and characteristic cytologic findings.^3^ This study was approved by the Ethics Committee of the Children's Hospital of Chongqing Medical University. All the patients' parents or legal guardians provided informed consent for using their medical records. Data were analyzed using SPSS (Statistical Product and Service Solutions). Descriptive analysis of different samples were carrried out by using percentages. Statistically significant differences between the data were evaluated using the Student's *t*‐test (*p* < 0.05).

## 3 RESULTS

### 3.1 Case presentation

Case 1. A 6 years and 2‐month‐old male with repeated fever and frequent convulsions for 12 days was admitted to our hospital and presented with convulsions and confusion accompanied by a fever heat peak of 40.3°C. The convulsion manifests as a focal origin of motor seizures with/without impaired perception and may progress to generalized tonic–clonic episodes. Physical examination showed red rashes all over the body. Meningeal irritation signs were absent, including neck stiffness and Kernig's sign. During hospitalization, a 1.2 × 1.5 cm lymph node was found in the left neck and palpable superficial lymph nodes were found in the right neck.

The patient's blood count, erythrocyte count, and hemoglobin decreased but the other indicators were not significantly abnormal, while C‐reactive protein and sedimentation increased considerably. The patient's two cerebrospinal fluid (CSF) biochemical detection, including protein, glucose, chlorine, and white blood cells, showed that these were within normal ranges.

Cranial CT and MRI showed no abnormalities. Video electroencephalogram (EEG) showed abnormal EEG. There were 17 seizures during the episodes, characterized by the right twitch of the mouth, a forward or left gaze of the eyes, right forearm clonus, and trembling of the lower limbs. The origin of synchronous EEG was changeable, mainly on the left side, and spread to the whole brain with high amplitude spiky slow waves and fast rhythm. The brain showed a background of 1–3.5 Hz activity in the interictal phase with a tight rhythm in the right central frontal region.

Due to high inflammatory indicators and suspected intracranial infection, he was administered intravenously (IV) with ceftizoxime sodium and acyclovir. The patient was given levetiracetam, sodium valproate, and oxepine combined with antiepileptic therapy because of persistent fever and repeated seizures. However, the treatment had poor effectiveness. Therefore, febrile infection‐related epilepsy syndrome (FIRES) of unknown cause was considered, and the number of episodes gradually decreased during hospitalization. Ultrasonography of the neck showed bilateral cervical lymph node enlargement. A biopsy of lymph nodes confirmed histiocytic necrotizing lymphadenitis. The patient's temperature had normalized after the diagnosis was established and the blood count and inflammatory parameters had returned to normal, so no hormones were administered. No seizures occurred for 27 days after onset but recurrent fever remained. The body temperature returned to normal 42 days after onset.

At discharge, the patient's body temperature was stable without seizures, and the oral administration of oxcarbazepine, sodium valproate, and levetiracetam continued for 7 months with no seizure recurrence.

Case 2. A 10‐year‐old female was admitted to the hospital due to right neck mass pain, fever for 20+ days, and headache for 4+ days. The patient was presented with neck mass pain, fever, and paroxysmal headache, which is mainly located in the bilateral temporal accompanied by vomiting and persistent aggravation. The patient's mother had a history of KFD. On physical examination, one lymph node with a diameter of 2–2.5 cm was palpable on the right neck and 2–3 lymph nodes with a diameter of 0.3–0.5 cm were palpable on the left. No meningeal irritation signs were found.

The patient's EEG and MRI were normal. The lumbar puncture examination showed elevated protein (0.61 g/L; normal range, 0.15–0.45 g/L) and white blood cell count (19 × 10^6/L; normal range, 0–8 × 10^6/L) with glucose and chlorine levels being normal.

Initially, the patient was considered to have an unexplained intracranial infection and was given cephalosporin antibiotics as anti‐infection therapy. The patient took a cervical lymph node biopsy that confirmed histiocytic necrotizing lymphadenitis. Based on the clinical manifestations and the CSF and cervical lymph node biopsy findings, the patient was diagnosed with KFD with aseptic meningitis. After anti‐infective treatment and mannitol were used to reduce cranial pressure, the body temperature was decreased and the fever interval was prolonged. Still, there was no significant relief from headache symptoms. Methylprednisolone was given by intravenous drip (60 mg per 12 h), and the symptoms of fever and headache were improved after 2 days. After 4 days of hormone use, WBC did not recover significantly and hormone doses were adjusted to 240 mg/d. After WBC recovered, the treatment was changed to oral methylprednisolone administration (20 mg twice daily).

The patient was discharged without fever or headache and continued to receive oral prednisone for 2 weeks after discharge and no recurrence occurred.

### 3.2 Literature review

#### 3.2.1 Demographics and clinical symptoms

Nineteen pediatric patients were identified, including two patients we presented (Table 1).^2^, ^4^, ^5^, ^6^, ^7^, ^8^, ^9^, ^10^, ^11^, ^12^, ^13^ Eight patients were males and eleven patients were females. The onset age ranges from 6 to 18 years (the median age was 14 and the interquartile range was 9–16). The clinical characteristics are summarized in Table 1. Sixteen patients were diagnosed with aseptic meningitis (84.1%, 16/19), one patient with brainstem encephalitis (5.3%, 1/19), one had brainstem encephalitis, one had febrile infection‐related epilepsy syndrome (5.3%, 1/19), and one patient was diagnosed with encephalopathy associated with KFD (5.3%, 1/19).

**TABLE 1:** The clinical features of central nervous system damage cases with Kikuchi–Fujimoto disease.

Date	Gender/age	CNS clinical manifestations						Interval (weeks)	CNS duration	CSF				EEG abnormality	Head CT/MRI abnormality	Hormone therapy	Outcome
		Headache	Confusion	Convulsions	Neck stiffness	Kernig's sign	Other manifestations			Pressure (mmH_2_O)	WBC (×10^6^/L)	Protein (mg/dl)	Glucose (mg/dl)				
1985^2^	F/16	+	‐	‐	+	+	‐	2	2	180	95^MC^	51	46	/	/	‐	C
1989^2^	M/13	+	‐	‐	+	+	‐	2	4	ND	1685^MC^	198	59	/	/	‐	C
1998^2^	F/8	+	‐	‐	+	+	‐	3	2	ND	49^L^	26	46	/	/	‐	C
1999^4^	F/14	+	+	‐	‐	‐	‐	3	ND	ND	32^MC^	48	68	/	/	+	C
2003^5^	F/17	+	+	‐	‐	+	‐	ND	ND	ND	19^ND^	160	54	+	‐	‐	C
2009^6^	F/13	+	‐	‐	‐	‐	‐	3	4	180	65^L^	28	56	/	+	‐	C
2014^7^	F/16	+	‐	‐	+	‐	Bilateral nystagmus and ataxia	ND	1	ND	ND	86	62	/	‐	‐	C
2014^8^	M/9	‐	‐	+	+	+	‐	3	2	>45	I^L^	77	ND	‐	+	+	S
2016^9^	F/15	‐	‐	+	+	+	Nystagmus, blepharospasm, ataxia	2	4	ND	45^L^	28	76	‐	+	+	C
2018^10^	M/12	+	+	‐	+	+	‐	3	2	ND	194^L^	312	52	/	+	+	C
2018^10^	M/17	+	‐	‐	‐	‐	Behavior disorder	2	1	ND	163^L^	333	42	+	+	+	C
2018^10^	F/15	+	‐	+	‐	‐	‐	1	ND	ND	87^L^	116	43	+	ND	+	C
2020^11^	M/18	+	+	+	‐	‐	‐	Simultaneously	ND	>300	454^MC^	400	N	/	+	+	C
2021^12^	M/9	+	‐	‐	‐	‐	‐	6	2	ND	100^L^	86	43	/	+	+	C
2021^12^	F/15	+	‐	+	‐	‐	‐	2	ND	ND	N	11	N	/	+	+	C
2021^12^	F/7	‐	+	+	‐	‐	Ataxia and status epilepticus	12	ND	ND	42^L^	22	ND	/	+	+	S
2022^13^	M/15	+	‐	‐	ND	ND	‐	ND	8	>30	70^L^	N	N	/	‐	+	C
Case 1	M/6	‐	+	+	‐	‐	‐	4	4	ND	1^ND^	19	53	+	‐	‐	C
Case 2	F/10	+	‐	‐	‐	‐	‐	2	1	ND	19^ND^	61	44	‐	+	+	C

*Note*: Interval, between enlarged lymph nodes and CNS symptoms; –, absent; +, present; /, not available.

Abbreviations: C, cure; CNS, central nervous system; CT, computed tomography; F, female; I, increase; L, lymphocytes dominant; M, male; MC, mononuclear cells dominant; MRI, magnetic resonance imaging; N, normal; ND, not described; R, relapse; R&C, cure after relapse; S, sequelae.

Headache (78.9%, 15/19), confusion (31.6%, 6/19), convulsions (31.6%, 6/19), neck stiffness (36.8%, 7/19), and positive Kernig's sign (36.8%, 7/19) were the most common symptoms and signs. Three patients had ataxia, two had bilateral nystagmus, and one had epileptic status. One patient had behavioral disturbances. The interval between onset and the symptoms of nervous system involvement was 1–12 weeks, and the duration was 1–12 weeks. Still, 1 case with neurological symptoms appeared before lymph node enlargement (Case 1 14 days).

#### 3.2.2 Investigations

Cerebrospinal fluid pressure was measured in 5 patients. In 3 patients, the CSF opening pressure was extremely high (60.0%, 3/5), cell count was elevated in 14 patients (73.7%, 14/19), 10 cases (52.6%, 10/19) were classified as predominantly lymphocytic, glucose was decreased in 4 patients (21.0%, 4/19), and protein was elevated in 12 patients (63.1%, 12/19). A total of 13 patients with pathogenic examinations, including antibody or PCR tests for Epstein–Barr Virus, Cytomegalovirus, Herpes simplex virus, and human herpesvirus, showed negative results.

Thirteen patients had cranial imaging descriptions, of which nine patients (69.2%) had cranial imaging changes involving the cerebellum, brainstem, temporal lobe, occipital lobe, pons, and leptomeninges enhancement. One patient had multiple diffusion restriction foci in a central perivascular distribution and central perivascular enhancement. Two patients had abnormal signals in the bilateral basal ganglia.

EEG was performed in 7 patients, and abnormal EEG was reported in 57.1% (4/7). Three patients had varying degrees of brain wave slowing, suggesting poor brain function. One patient was suggestive of encephalopathy with a few seizure potentials, and the latter two showed abnormal discharge of focal origin. In one case, repeated seizures occurred in episodes (Case 1).

Pathological biopsy of lymph nodes was performed and all 19 cases were diagnosed with HNL.

#### 3.2.3 Treatment and outcome

A total of 12 out of 19 patients (63.2%) received steroid therapy. One patient was treated with phenytoin sodium in combination with antituberculosis, and one patient was treated with antiepilepsy with intravenous gammaglobulin infusion.

A total of 17 out of 19 patients (89.4%) completely recovered but two patients (10.6%) had neurological sequelae, including some neurocognitive, neuropsychiatric, and behavioral sequelae that persisted.

## 4 DISCUSSION

KFD with neurological symptoms is rare. There have been 33 reported cases of KFD complicated with central nervous system injury in adults.^13^, ^14^, ^15^, ^16^, ^17^, ^18^ Only a few cases of KFD with central nervous system damage have been reported in children. Studies on adult KFD patients with central nervous system damage have suggested that males may have a gender advantage. The proportion of males in some pediatric KFD study cohorts was slightly higher than that of females.^19^ However, in our pediatric literature cohort, 8/19 (42.1%) were males, which is inconsistent with the situation reported in the literature. It may be related to racial differences and the small number of cases reported in our study. In total, 68.4% of our patients were between 12 and 18 years old, while 31.6% were between 6 and 11 years old, with a median age of 14. It is older than the mean ages in KFD pediatric studies and has ranged from 11 to 13.2 years old.^19^, ^20^ Meanwhile, in adult patients, the age range was 19–57 with a median age of 28 years old, and most of the onset age was less than 30 years (70%).^13^, ^14^, ^15^, ^16^, ^17^, ^18^

The clinical manifestations of KFD involving the central nervous system are complex and varied. Aseptic meningitis is KFD's most common central nervous system complication,^21^ similar to adult patients. Our pediatric literature cohort's main presentation was headache (78.9% 15/19). Other symptoms, such as ataxia (15.8% 3/19) and behavioral disorders (6.7% 1/19) were also reported in adults. However, clinical manifestations of convulsive episodes have rarely been reported in adults. Repeated seizures as the primary clinical manifestations of the nervous system are seldom reported, and pediatric patients are prone to missed diagnoses. In our pediatric literature cohort, we describe two cases of KFD with central nervous system damage, one of which presented with FIRES (FIRES refers to fever starting between 2 weeks and 24 h before the onset of refractory status epilepticus, with or without fever at the beginning of status epilepticus).^22^ These children are easily misdiagnosed with refractory epilepsy due to the poor effect of antiepileptic drugs. For pediatric patients who are presented with FIRES, especially those with a fever of unknown origin, the possibility of KFD should be considered, and lymph node‐related screening is necessary. Patients with necrotic lymphadenitis complicated with central nervous system damage may be misdiagnosed as intracranial infection of unknown etiology if the early clinical manifestations are only fever and headache without lymph node enlargement. In addition, detailed lymph node examination and related examinations may be necessary for patients with suspected intracranial infection of unknown etiology who have failed or have not responded well to anti‐infective therapy.

In our literature cohort, the number of CSF cells and protein in most patients increased significantly, and a few had a slight decrease in CSF glucose. These changes were nonspecific and similar to those reported in adult cases. It was a routine examination to rule out brain diseases caused by common pathogen infections.

In our case series, cranial imaging changes involve the cerebellum, brainstem, temporal lobe, occipital lobe, and pons consistent with the condition reported in the literature review.^13^ There were also midbrain and gray matter involvement in our case cohort, and patients with midbrain lesions showed nystagmus, blepharospasm, ataxia, and focal symptoms corresponding to the imaging abnormalities. Patients with gray matter lesions had neurological sequelae and some cognitive, mental, and behavioral symptoms persisted, which had not been described in the previous literature.

The EEG perfection rate was low in both adult and pediatric patients. In our literature cohort, 3 patients presented varying degrees of background wave slowing, suggesting brain function impairment. In one of our reported cases, the EEG of the patient showed multiple seizures with focal origin spreading to the entire brain, predominating on the left side. For KFD patients complicated with central nervous system damage, it is necessary to improve EEG combined with cranial MRI/CT to evaluate brain function and organic brain lesions.

Although KFD is mostly self‐limited, steroid administered as immune modulators in patients suffering from prolonged fever has been reported to shorten the clinical course of the disease.^20^, ^23^ In our study, the clinical duration of not taking steroid therapy ranged from 1 to 12 weeks (the median was 4 and interquartile range was 1.75–6), and those who received steroid therapy ranged from 1 to 8 weeks (the median was 2 and interquartile range was 1.25–3.5), *p* > 0.05, showing no significant difference between them. It is not consistent with the situation reported in the literature. Further study and analysis of whether steroids can shorten the clinical course of KFD complicated with central nervous system injury is needed.

KFD studies in children showed a high recurrence rate of 42.4%.^19^, ^24^ The presentation of recurrence is usually similar to the initial fever and cervical lymphadenopathy.^25^ However, recurrence of central nervous system damage is rarely reported, and there were no recurrence cases in our study, which is inconsistent with the high recurrence rate reported in children. Since the pathogenesis of KFD is still unclear, whether this difference is related to the small number of cases studied, race, and central nervous system involvement still needs further study. Two patients had neurological sequelae, which may be related to the patient's age and the site of brain injury indicated by performing a head MRI.

## 5 CONCLUSION

Our study delineates the clinical manifestations of KFD in children with central nervous system damage. Aseptic meningitis is the most common complication, presenting mainly with headaches. We also report a rare case with repeated seizures as the initial presentation. Those patients are prone to misdiagnosis, and relevant screening is necessary. KFD is a self‐limiting disease but has a high recurrence rate in children. Although no patient relapsed in this study, irreversible damage to brain function may have been caused, and follow‐up is still necessary.

## AUTHOR CONTRIBUTIONS

Li Jiang and Yan Jiang designed the study. Juan Wang, Chengbing Tan, Min Cheng, and Yan Jiang collected the patient's clinical information. Yuyao Huang performed the statistical analysis and drafted the first version of the manuscript. All authors read and approved the final manuscript. All authors have complete access to the study data supporting the publication and agree to the submission policies.

## CONFLICT OF INTEREST STATEMENT

All authors declare no conflict of interest.

## ETHICS STATEMENT

This study was approved by the Ethics Committee of the Children's Hospital of Chongqing Medical University. All the patients' parents or legal guardians provided informed consent for using their medical records.

## Data Availability

Data sharing is not applicable to this article as no new data were created or analyzed in this study.
